# From Sensors to Digital Twins toward an Iterative Approach for Existing Manufacturing Systems

**DOI:** 10.3390/s24051434

**Published:** 2024-02-23

**Authors:** Dimitri Renard, Ramla Saddem, David Annebicque, Bernard Riera

**Affiliations:** 1 Centre de Recherche en Sciences et Technologies de l’Information et de la Communication, University of Reims Champagne-Ardenne, 51100 Reims, France; dimitri.renard@univ-reims.fr (D.R.); ramla.saddem@univ-reims.fr (R.S.); david.annebicque@univ-reims.fr (D.A.); 2 Prosyst, 59300 Valenciennes, France

**Keywords:** digital twin, manufacturing, data collection, IoT, scaling

## Abstract

Digital twin technology is a highly valued asset in the manufacturing sector, with its unique capability to bridge the gap between the physical and virtual parts. The impact of the rapid increase in this technology is based on the collection of real-world data, its standardization, and its widespread deployment on an existing manufacturing system. This encompasses sensor values, PLC internal states, and IoT, as well as how the means of linking these data with their digital counterparts. It is challenging to implement digital twins on a large scale due to the heterogeneity of protocols and data structuring of subsystems. To facilitate the integration of the digital twin into existing manufacturing architectures, we propose in this paper a framework that enables the deployment of scalable digital twins from sensors to services of digital twins in an iterative manner.

## 1. Introduction

The manufacturing industry is rapidly transforming into a new era, Industry 4.0. Data and their analyses are the main components of this industrial revolution that we are experiencing. Artificial intelligence, connectivity, Digital Twin (DTw), and intelligent inter-issues obtain data from the real world. DTw is a technology based on this connectivity between the real and virtual worlds [1,2] whereby the virtual is a vision of its real part to increase its capabilities. Different devices have different data refresh rates, power, and communication protocols. This diversity in the world of Operational Technology (OT) makes it complex. Feeding a DTw with data from the physical world is a real challenge requiring expertise from OT to Information Technology (IT) [3]. This challenge is very difficult when the system already exists. Deploying a DTw therefore has a significant cost to design and deploy it, and also to maintain it [4]. This is because collection, models, and analytics must be constantly updated with real-world developments. Duplicating a DTw facilitates the return on investment and the development of this technology, which is expensive to implement [5]. Data collection is the pivot between the two worlds. The available data must allow the creation of standard services and, conversely, the lack of data must allow the standardization of their measurement and collection [6]. The difficulty of large-scale deployment of standardized data collection in the manufacturing sector is slowing down the deployment of a first generation of DTws in existing systems. The objective of this article is to propose a framework for the development and large-scale deployment of an evolutive DTw in the manufacturing domain.

Section 2 provides a concise state of the art of the DTw concept. The purpose of this overview is to show the main characteristics of DTw, which are applicable in a wide range of industries. This overview also aims to show the potential benefits of DTw, the link with simulation and IoT technologies, as well as the need for methodologies to define a DTw project.

Section 3 aims to show the challenges of implementing communication between the real and virtual worlds even more in the context where the system already exists. This part also shows the importance of contextualizing data to provide a maximum understanding of them in the virtual world of DTw. To meet these challenges, the OPC-UA technology, which is increasingly standard in the manufacturing world for its communication protocol, also allows the contextualization of data and the provision of information models.

Section 4 provides a framework for iterative DTw design on legacy systems. Services self-configure through the provision of contextualization data, and the addition of a sensor instantiates new services. As self-configuring sensors and services are implemented, DTw will become more and more mature.

We conclude with a discussion explaining the opportunities and limitations of this iterative approach and conclusion.

## 2. Digital Twin Overview

This section is intended to provide an overview of the concept of DTws, and the advancements in research on the subject, particularly in the manufacturing sector. It will also try to show the difficulties in defining and implementing a DTw for its need to limit the risks of these projects.

### 2.1. The Basics of the Concept of a Digital Twin

The basis of the concept of DTw is in the following two definitions, that of Grieves [1] “The Digital Twin concept model […] contains three main parts: (a) physical products in Real Space, (b) virtual products in Virtual Space, and (c) the connections of data and information that ties the virtual and real products together.”, and Glaessgen and Stargel [7], “Digital twin is an integrated multi-physics, multi-scale, probabilistic simulation of a complex product and uses the best available physical models, sensor updates, etc., to mirror the life of its corresponding twin.” The DTw is, from its earliest definitions, the mirror of its real physical twin. The complexity of the implementation of this technology, its youth, and its promised perception have caused a distorted or incomplete image of the actual technology, which is the DTw. A more recent definition of DTw is given by Tao et al. in 2018 [8,9], “[…A] complete DT [Digital Twin] should include five parts: physical part, virtual part, connection, data, and service.” It takes up Grieve’s [1] by clearly introducing the notions of data and service; the five-dimensional DTw can be integrated. In order to characterize the DTw, Jones et al. synthesized the work and identified 12 themes that describe and characterize the DTw [10]: Physical/Virtual Entity, Physical/Virtual Environment, Physical/Virtual Process, Fidelity, Status, Parameters, Twinning Frequency, and Physical-to-Virtual/Virtual-to-Physical Connection. Many other syntheses on the DTw have been produced in recent years, clarifying this complex notion more and more [11,12,13,14,15,16,17,18,19]. They bring together real-life use cases that make it easier to understand the possibilities of DTw as well as the technologies currently available to implement its DTw in its environment. A new generalist standard named “Digital twin concepts and terminology” was published at the end of 2023 [20]. It defines the DTw as the “digital representation of a target entity with data connections that enable convergence between the physical and digital states at an appropriate rate of synchronization”. This is a very broad definition of DTw, but it has all the essential concepts that make up a DTw.

### 2.2. Definitions for Each Domain

As DTw is a technology that can be used in a variety of fields, definitions that are more specific to business areas are proposed, such as those focused on the product life cycle [21,22], such as Stark’s et al. definition in 2019 [23], or the manufacture-oriented one, such as the Negrie et al. definition in 2017 [24]. The high demand for implementations in the manufacturing sector has led to the development of the ISO 23247 standard to standardize the DTw for these requirements [25]. The DTw is divided into four blocks: User, Core, Data collection and Device control, and Cross-System. Interfaces with humans are an integral part of this standard framework, which could potentially shift toward the symbiosis between humans and the DTw. From measurement by sensors to ergonomic data for use, many technological blocks must be developed and implemented. In addition, human DTw interfaces must be adapted to the user’s needs. Figure 1 of [26] shows the interactions between DTws, based on the granularity of observable manufacturing elements (OMEs) and their correspondence with the different requirements within the company.

These definitions enable us to better understand the possibilities of DTw in each field, as well as how to implement it in its activities. However, they also have the potential to diverge the understanding of the DTw by limiting its possibilities to those possible in their field (see Table 1).

### 2.3. Digital Twin Gain

The first two sub-parts introduce the concept of DTw and its multiple definitions that are specialized in a field of application. However, these definitions do not give any indication of the different purposes that DTws may have.

The purpose of DTw is to give a certain omniscience to its users, making operations less expensive because they are more efficient. It also offers material gains by providing predictive maintenance services, the possibility of simulating certain measurements, or increasing the performance of the system. In other words, the DTw is a tool that offers easy access to enriched information to all its users, eliminating many unnecessary human and material costs [28].

**Figure 2:**
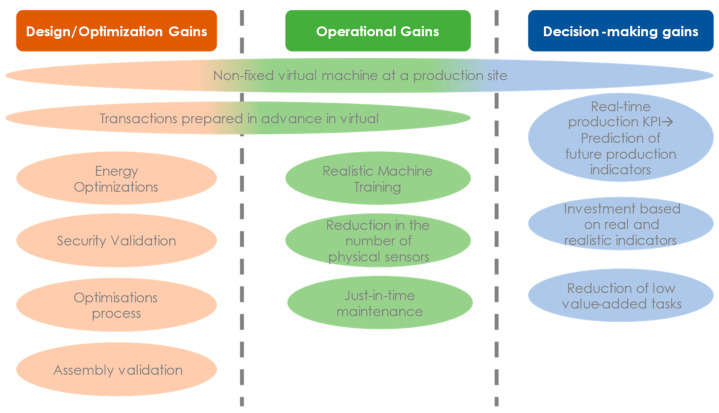
Digital Twin Possibilities.

A maintenance operation on a sensor can be recommended by a DTw department, and technicians can prepare it via the planning and visualization service. The gains in this example are multiple: the sensor is only changed when it starts to malfunction; and accessibility operations and tools will have been planned before the intervention, which optimizes the efficiency of human operations and reduces machine productivity losses. The DTw can also be used to predict the future behavior of the system by simulating the future behavior of the system based on the real situation, to test parameter optimizations by simulation, to validate them, and to provide feedback on the control accordingly. The gains are not only at the operational level but also at the decision-making level, facilitating the analysis of performance, quality, and energy consumption. Indeed, a production line DTw can be accessed from a machine as well as from a centralized service. Thus, the decisions are made based on data based on reality and not only on theoretical projections (Figure 2). To be able to obtain its possibilities from DTw, simulation and IoT technologies will be part of a large part of DTws.

### 2.4. Digital Twin and Simulations

As we have seen in the previous parts, the DTw is more than just a simulation. Indeed, it is the link with reality that makes it unique and powerful. It is an essential part of the standard DTw definition [20,25]. A simulation is not a DTw, as it is not connected to the real system. However, DTws can have services that utilize simulations. These can be visual with a three-dimensional representation, but they may not have a graphical interface. Indeed, the models used can be of any kind. A simulation cannot be a DTw. However, the DTw can use simulations. The distinction between these two concepts is necessary to address the need for the right technology. The opening up of simulation services executed and initialized according to parameters captured from the real world appears to be an important way for exploration. Indeed, the applications of the simulations can then be used as close as possible to the system in operation to predict failures or even propose optimizations. Simulation models developed in the design phase can continue their life phase to be used in a DTw service. DTw and simulations are not identical, but the two concepts are intimately connected.

### 2.5. Digital Twin and IoT

In the manufacturing world, production equipment has many measuring points necessary for control. However, to be able to integrate services that respond to the system environment, it is often necessary to increase the measurement capabilities of the real system, even in the manufacturing industry. The concentration of all its data in the control system is a result of the burden of the control system. IoT sensors are therefore responsible for collecting data that are not necessary for control but have a potential for optimization analyses. IoT technologies facilitate data collection by offering more standards and capabilities directly on sensors [29,30,31]. Thus, these sensors allow a broadening of the measurements that can be made to understand the system in its environment. It is not necessary for them to be added to the DTw, but they enable a possible increase in its capabilities if services utilize them [32]. Another aspect between these two concepts is the distribution of the first data processing. Indeed, an IoT layer with enough computing and storage power can consolidate with a certain intelligence the data directly from the shopfloor [33]. This is a key point in the possibilities of DTws in view of the quantities of data collected and to be transmitted and stored beyond the production networks.

### 2.6. Define Your Digital Twin

Agrawal et al. propose a framework to assist in defining its DTw [34]. It divides the DTw into four elements (Observation, Analysis, Decision, and Execution). Their framework is composed of a two-dimensional graph: the element and the level of automation. It replaces the objective of the DTw by providing a tool for reflection on its anticipated objectives. Iteratively, DTw projects are designed with a reflection on the needs, constraints, and expectations. On an existing system, the integration of a DTw must be integrated as seamlessly as possible to disrupt production as little as possible. This means that the optimal balance between needs and effort/costs can be defined more easily [34]. Analysis, decision making, and execution are only possible through information. The observation part is therefore essential [35] and often overlooked in the DTw. A DTw project that is most suitable for its needs and constraints is a difficult task that still requires new methods. The ISO 9001 [36] model approach is an opening to reflect on all DTw design issues [37]. The creation of a DTw from a library of components that is gradually developed, and reusable, seems to be a way to achieve more massive deployments across an entire shop floor [38]. As the age of DTw deployments is still in its infancy, it is important to understand the significant risk of deployment over a large area. Observation is often forgotten in the design idea of the DTw even though it is one of its main features.

### 2.7. Digital Twin Maturity

**Figure 3:**
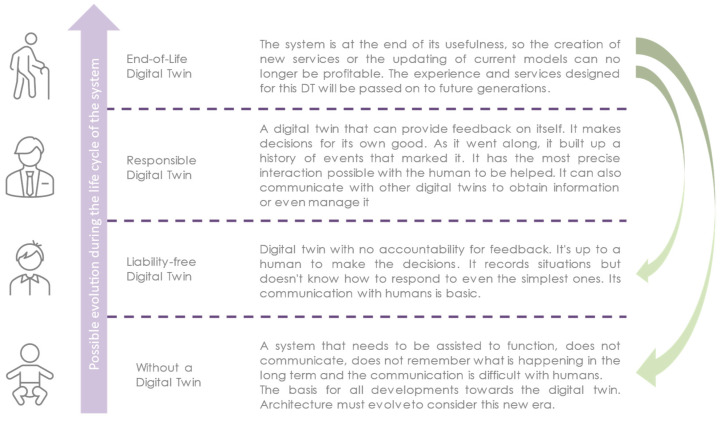
Digital Twin maturity.

Private and public research laboratories are designing and implementing proofs of concept and frameworks on this technology. The reality on the ground can hinder the relevance of these tools [19]. However, the maturation of the DTw evolves as it encounters this technology. The constraints of the application domains will be incorporated to create an implementable DTw. The DTw is composed of mechanical and control architectures. It is difficult to modify this architecture in production during the life cycle of the production line. DTw grows over time, as do the different services that aggregate with projects. It increases these capabilities more and more iteratively. Thus, the gains of the DTw are reduced by the project risks [39]. It also learns, during its life cycle, about different models of settings, normal behavior, failures, etc. They can be used to monitor the system more accurately. DTws can be enhanced by their ability to communicate with other DTws to obtain information. These capabilities could even be from a management perspective with higher-level services. At the end of the system’s existence, the DTw is also at the end of its life. The experience gained with the creation of this DTw, as well as the limitations encountered, must be considered for the design of the next DTw generation. It can also be used to store the real system for the future. Figure 3 summarizes how a DTw can evolve during its life cycle. However, the DTw can reach the conclusion of its life without having passed the various levels of maturity. Nevertheless, experience must be retained to improve future versions of the system. The real and virtual parts of the DTw are not as independent as the DTw suggests. They complement each other to form an integrated system with more control over themself as well as more communication capabilities.

### 2.8. Overview Discussion

This overview of the state of the art of DTw made it possible to show the concept in its generality. The different definitions, presented in this overview, allow the main characteristics of DTw to be observed. The evolution of definitions is very rapid, and more and more standards define DTw. Our state of the art is concise and oriented toward the applicability of the DTw concept, particularly in the manufacturing world. This overview provides a DTw maturity model to help understand the diversity of different DTws that can change over time. The investment of time and money to achieve a retroactive DTw is colossal, from design to maintenance. This is especially the case when the system is in production and has not been designed with DTw implementation constraints in mind. Rigor in the definition of the need and the design methodology of the DTw is therefore necessary. The communication between the real and virtual parts of the DTw must be able to keep up with the changes in the maturity of the DTw. In the next Section, we will look at the practical issues of this part of the DTw when implementing it on an existing production system.

## 3. Digital Twin Connection between Real and Virtual

The purpose of this Section is to illustrate the aspects of the connection between the real and the virtual parts in the manufacturing industry. Indeed, this connectivity between the real and virtual worlds is the hallmark of the DTw. It is therefore essential for its deployment. Data collection in sensor-equipped service workshops takes place at a level above that of the order. Interoperability and knowledge of equipment are the first points. Then, the contextualization of the data collected is also just as important. The OPC-UA brings these aspects together in an open technology that is increasingly standard not only in the field of manufacturing but also in research.

### 3.1. Interoperability

Interoperability between the company’s various systems is at the heart of Industry 4.0 issues. Silo architecture between trades must be deconstructed to facilitate the transmission of data. Reference architectures [40] have therefore been created as the Reference Architectural Model Industry 4.0 (RAMI 4.0) or the Industrial Internet Reference Architecture (IIRA). Depending on the life cycle of the product and the location of the company, the possibilities for interoperability differ. This interoperability encounters major challenges in the field of operation technologies. In addition, the communication protocols of the equipment in the field are varied and often proprietary. Additionally, the communication performance of some protocols in equipment is not yet complete (e.g., OPC-UA embedded servers). As a result, they are sometimes not up to the standard of the proprietary protocols available. In some cases, interoperability is not possible at the lowest levels. It is then up to the top layer to take over to ensure this. The construction of an architecture from the sensor to the DTw is therefore essential. The standardization of communication protocols should facilitate this issue of connection to data. However, this process is going to be a lengthy one and will be a crucial element in the OT world for many years to come. Implementing a scalable DTw involves considering the evolution of technologies during the system’s life cycle. Thus, interoperability is a challenge for the implementation of a DTw in the architectures of current production lines but will also be so in the future between several generations of DTws.

### 3.2. Contextualization

**Figure 4:**
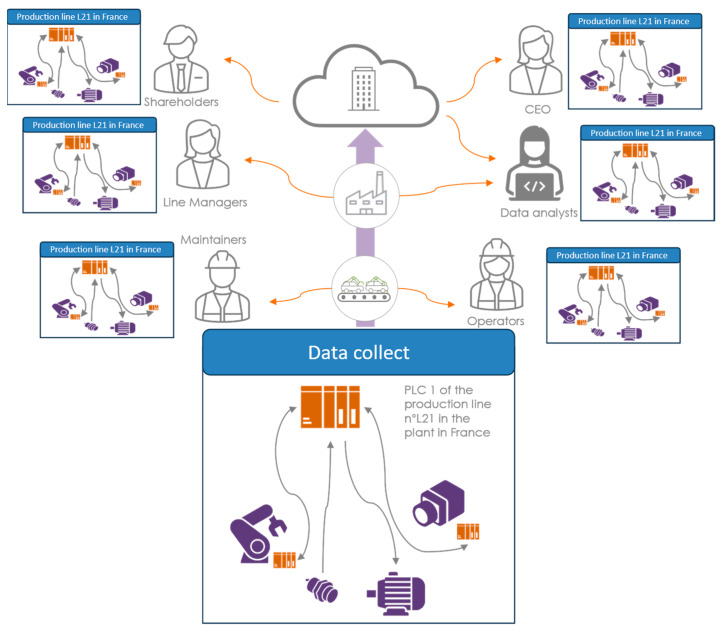
Contextualized data for better analysis at all levels of the company.

The value collected from the real world does not yet have a meaning associated with it. It is this meaning that makes it contextualized. All the metadata around the data then allow this to be understood by those who consume them. This contextualization can take the form of units of measurement (kg, m, s, ms^−1^, W, etc.), a hierarchy of components (the sensor X belonging to robot X in line X of factory X, etc.), type of process, etc. From the perspective of open data architecture in the company and interoperability, this contextualization is essential to maintaining consistency and understanding between the various trades. Thus, the data are aggregated as they move through the levels of the company with the richness of their metadata so that they can be used in the best possible manner [41]. Figure 4 illustrates the potential impact of contextualizing data from the moment they are collected for all the company’s stakeholders, even beyond their use in a DTw. This is because each core business receives a virtual representation of the system perception and not a raw list of measurements. The contextualization of the data brings the system closer to each person who needs to analyze them. These metadata, beyond being utilized for human understanding and data analysis, are also a source of information for algorithms and models. It is possible to find the same and consistent data across multiple production sites, giving us a bigger data set. The deployment of services can also be increased by contextualization metadata by self-configuring with the necessary data.

### 3.3. OPC-UA

The Open Platform Communication Unified Architecture (OPC-UA) is a secure industrial protocol but also a data space for industrial systems. The OPC Foundation has been established, maintained, and improved since 2008. It is the result of the unification of OPC Data Access, OPC Historical Data Access, and OPC Alarms and Events, as well as the distancing of itself from Microsoft’s proprietary COM and DCOM protocols. IEC 62541 [42], RAMI 4.0, and IIRA utilize OPC UA as a communication protocol for industrial assets. The OPC-UA includes the possibility of opting for a publish/subscribe mode. A popular way to implement this mode is to use OPC-UA through the MQTT protocol. This is called “OPC-UA over MQTT”. This openness allows for greater interoperability with consumers in the IT sector. The security measures implemented in this communication standard are commensurate with potential attacks. This is a key point in the possibilities of opening more possibilities for secure exposures of the data collected. The benefits of this technology are being felt in the manufacturing and research sectors, where implementations are becoming increasingly widespread.

The use of an open and royalty-free protocol on a large scale will facilitate not only the interoperability of field equipment, but also the more widespread standardization of data collection on it.

## 4. Integrated Approach from Sensor to Digital Twin

**Figure 5:**
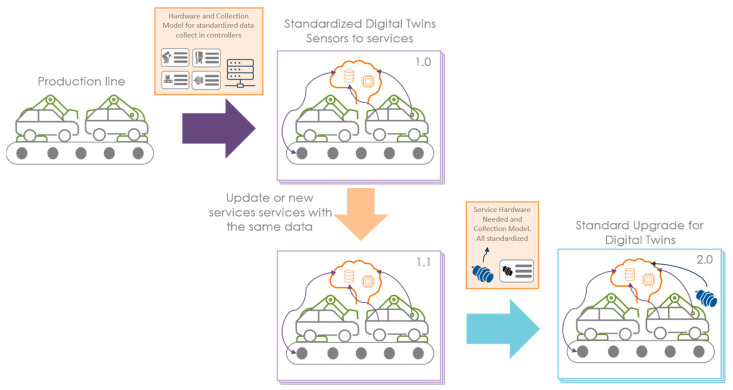
Sensors to Digital Twin evolutions.

There is a growing demand for DTws to improve the performance of factories worldwide [43]. However, the DTw takes time to develop and must be maintained continuously. Our integrated approach from sensor to DTw aims to make it rapidly deployable and scalable in today’s industrial environment. However, it also expands the possibility of evolution over time to capitalize on experiences, and allows the segmentation of projects to facilitate the allocation of associated budgets. Iteratively, in the manner of the spiral model, with data collection as a pivot between the two worlds, our approach proposes a scalable DTw from the sensor to its services (Figure 5). This architecture will be detailed in the different subsections.

### 4.1. First Data Collection for Digital Twin

The DTw’s data collection is the hub for various services. What data are available on the different controllers, and in which am I interested? Are they accessible in a standardized manner? The right granularity of data collection must be determined to provide its DTw while considering the difficulty in deploying this data collection brick on a large scale.

#### 4.1.1. What Is the Need for Data Collection?

The various controllers on the production lines are full of data. They can be of various types, such as continuous, discrete values, images originating from a capture of the real world, or states within the system itself. Some of these data, which are considered essential for the overall control of the system, are gathered by a higher-level controller coordinating its various equipment. Depending on the need, the granularity of data collection cannot be the same. Indeed, depending on the constraints of the network architecture, the refresh rates in the “aggregator” controller, the communication capabilities of the equipment, and the “aggregator” controller, the choice of the collection method is highly constrained. The need for a use case can guide the granularity of data collection so that it can best be addressed. However, performing data collection guided solely by the use case does not open the doors to a diversity of services. The DTw can therefore be designed unitarily with this approach, but it is difficult to replicate because it is driven by the use case. A hybrid approach based on a use case, but also on the scalability of service options, opens the possibility of large-scale deployment. Service projects are developed based on identified needs and available data.

Defining an initial need and the desired measures to try to solve it is the first phase of building a scalable DTw. It makes it possible to de-risk access to desired values and to initiate potential connectivity issues in the factory.

#### 4.1.2. Granularity Compatible with Standardization

However, the purposes of data collection are manifold, and the demands for data for production lines are high. The need is therefore to provide data on the equipment that is available in factories as easily as possible to improve their profits and limit their costs and losses. Choosing a granularity that is found time and time again in your company opens possibilities for standardization, both in terms of data collection and in all the services using standardized data at the level of your company. This is the second phase of the DTw’s connectivity design that is destined to be instantiated multiple times. Indeed, it is a phase of retreat to orient the following phases of the methodology toward more openness and, therefore, toward more potential gains. Thus, the granularity can be located at the level of a concentrating PLC as well as on the controller of a robot or a camera, depending on your case. This granularity must be sought with the use cases available, but also with the difficulty in finding a standardization for the equipment. This task of determining the data to be collected is complex. With the OPC-UA standard, companion specifications have been introduced for different equipment and areas of application. The purpose of this data collection is to establish a general standard for data collection by type of equipment or by field of application. However, it does not currently meet the needs of everyone. Thus, basing oneself on its specifications seems to be relevant. However, in some cases, it is necessary to adapt them for generalization in one’s own environment and specific needs. Furthermore, the development of these information models is rapidly evolving, which does not guarantee future compatibility with third-party applications.

#### 4.1.3. Collection and Its Limitations

Data collection is subject to technical limitations. Additionally, the collection of data from the various sensors present on the production line through the controllers utilizes part of their computing power. Hardware solutions exist to collect data without relying on control. However, this approach requires human intervention in the physical system, which significantly complicates the scalability of standardized data collection. This short-circuiting of data collection out of the controller does not include any direct feedback to the control. Depending on the capabilities, data collection can be driven by a standard view of it on a controller but can also be configured according to the associated services. However, in both cases, the data are collected only once on the controller and can be utilized by several services in the company.

The programming of the PLCs is a major limitation of data collection. Furthermore, the lack of control design methodology and the differences in the implementation of the ISO 61131-3 [44] standard between the different brands of PLC make it more difficult to collect data automatically. Some groups require a programming standard for themselves or their integrators in order to obtain standardized control of the PLCs in their factories [45]. However, this approach is not standardized, which only allows the most powerful groups to utilize it.

The contextualized data collected on the production lines are sensitive to the company. Furthermore, these data can be used by competing companies or as pressure during negotiations. Thus, data collection must consider this issue of cybersecurity. Additionally, in the event of feedback on controllers, this security vigilance must be increased due to the risks involved in the event of an attack. A secure communication protocol is the first step to take. However, the OT world is moving at a much slower pace on the renewal of technologies due to the longevity of the installations and the reliability required of them. It is through architecture that protections are and have been deployed to protect production lines from cyberattacks. We therefore find a segmentation of networks (by line, by zone, etc.) in manufacturing facilities. This protective architecture limits the possibilities of holistic access to controllers throughout a plant. To be able to escalate the data securely, a secure gateway to the higher levels of the company is then necessary. The OPC-UA protocol allows data to be grouped together in an address space that is securely accessible to authorized individuals. The standard also offers the possibility of a role with different rights on the server. Thus, the sensitive data collected on its production lines can be transmitted only to the authorized persons who need them.

### 4.2. Data Enrichment

The data collected from the various controllers and IoT devices in the factory must be enriched so that they can be understood, stored, exploited, etc. Mechanisms for contextualizing, aggregating, and consolidating data make it possible to increase the amount of data collected. However, the DTw is dynamic, requiring the addition of new measures and temporary data collections. The data collection part must be modular to enrich, in multiple ways, the data on which the DTw services capitalize. This data enrichment phase is carried out in parallel with the two previous ones to obtain data collection models that meet the needs that are as minimally intrusive as possible in the order.

#### 4.2.1. Contextualization of Data

Making decisions or analyzing raw values without any information about their representation is impossible. Simple floats, for example, cannot be understood without a connection to reality. Contextualizing data is the link between the user and the real world. This is all the information needed to understand a variable. Several possibilities can be implemented to convey this contextualization. The first is the self-collection of data to locate the end-to-end chain between the sensor and collected values. However, this “manual” method has limitations due to the skills required to carry out the project from the beginning to the end, as well as the possibilities of scalability and the possible saturation of the collection on the APIs. Computer approaches are therefore to be preferred to transmit this information around the values. The name of the variable can allow this contextualization in the different strata of the company. Thus, the current of axis 2 of the pick and place robot of production line 3 of the French factory can be found in the name of the variable. This solution is easily implementable and understandable for humans. However, variable names can be limited in size and require string operations to rebuild the variable tree. One technological solution to address this need for contextualization is the OPC UA. This technology includes an address space that allows the contextualization of the data, as well as a secure exchange protocol and openings via MQTT. This opening in MQTT is also standardized to be able to transmit contextualization through this protocol.

#### 4.2.2. Data Consolidation and Aggregation

The data collected, contextualized, and harvested from the lower layers of the factory are increasingly important with this transition to Industry 4.0. The collected data are directly put into a single silo and fed back to the cloud (Figure 4). It is with this immense lack of data that data scientists query to carry out their analyses. However, transporting and storing this mass of data comes at a cost. It is as much financial, in terms of the storage and retrieval of its data, as it is environmental. The governance of these data is also an important factor to consider. Consolidating data between different levels of the company partly limits this massive data transport between production line controllers and the cloud, as well as the resulting storage.

The consolidation of the data also makes it possible to carry out the first processing in a standardized and distributed way at each point of collection. In this way, the pre-processed and contextualized data can be distributed directly to the line operators. The processing of the collected data at the factory or cloud level involves a significant delay in having a timely response to the needs of the operator on site. Thus, preprocessing compresses the data to be transmitted to the higher levels of the company by distributing the computational load over the different collection points, and it also allows the synthesis of data in real time for operators.

**Figure 6:**
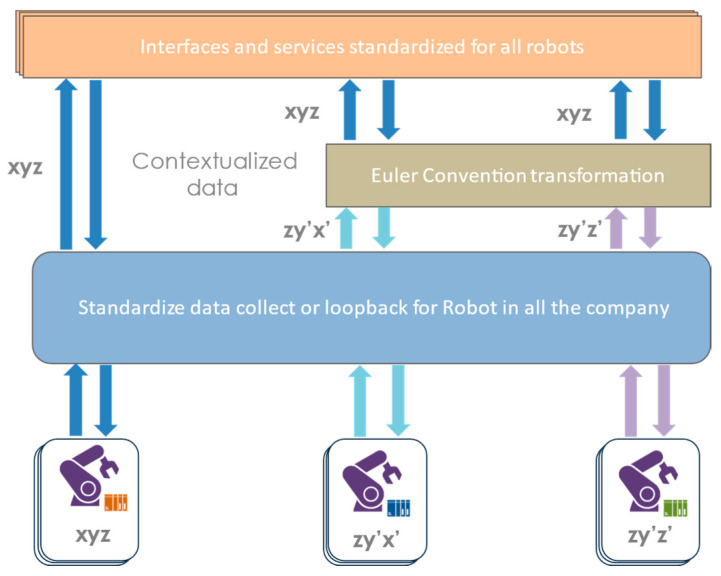
Data pre-processing robot example.

In the example in Figure 6, data collection between different robots can be different for different brands. However, it is possible to know the convention (e.g., Euler conventions) used and pre-process the data to expose them in a standardized way to analysts and to the various interfaces and services. In addition, each manufacturer has different connectors for their robots. Standardizing data collection makes it possible to find the same information for all robots, but also to pre-map the collection addresses in the controllers. Deploying a new data collection on one of its robots is then almost automatic. Indeed, instantiating a collection on a robot is the same as defining what defines the robot, such as its brand, its IP address, etc. The mapping work no longer needs to be performed. It is the same with the upper level of interfaces and services that can have automatic reconfiguration behaviors depending on the equipment or type of data collected. The standardized encapsulation of the technical aspects of data collection will facilitate the implementation of the data collection and processing flow from the workshops for different users.

#### 4.2.3. Modular Collection for Digital Twin Separates from the Control

**Figure 7:**
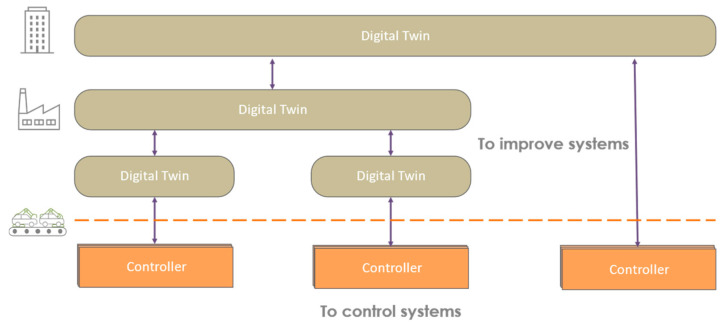
Controller and Digital Twin.

Data collection needs to evolve over time and different projects around the production line. Modular data collection is therefore preferable. However, the modularity of the collection should not be achieved by diversifying it. Considering the versioning of data collection models is the first solution to this modularity. It allows you to update the data collected not only for your new needs but also for your interfaces and all the associated services. In this way, the associated services can be versioned and tested globally before deployment on premises. This modularity of the collection deployment can be compromised if part of the control is carried out by the collection system. This is why the DTw seems to be, in principle, not essential for control. It optimizes the system, makes it more resilient, and makes it easier to understand while remaining independent (Figure 7). Thus, it can be scaled with more flexibility than controllers. However, this flexibility is limited by the architecture and interfaces of the controllers to obtain the optimizations from the DTw.

### 4.3. Evolution and Other Deployments of Digital Twin

**Figure 8:**
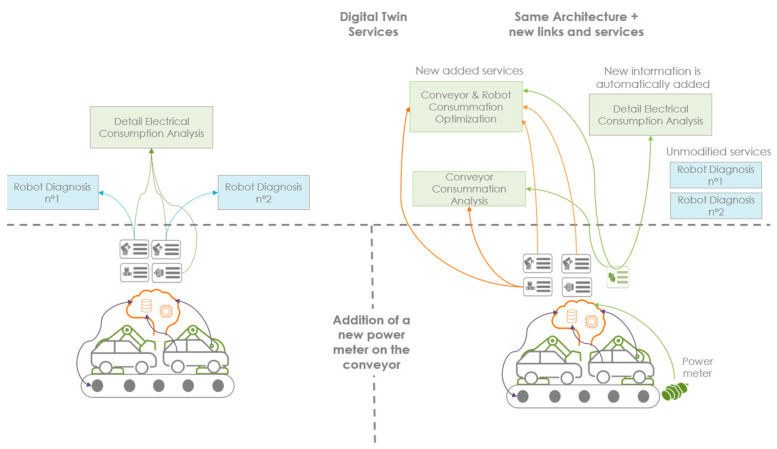
Iterative Digital Twin.

DTw projects are still largely unitary, with many manual operations. Facilitating the larger-scale deployment of the DTw is therefore a necessity for the industry to accelerate their deployments. Furthermore, the standardization of the DTw will also result in a better acceptance of the technology, with gains in production but also in the accessibility of data by operators. This is the last phase of the methodology to duplicate or evolve the DTw created in the upstream phases. Figure 8 shows what the framework produces when you design a new service and when you integrate new standardized sensors into the architecture. The aim of this section is to demonstrate that this framework offers the opportunity to deploy in existing systems iterative DTws.

#### 4.3.1. New Services and Intelligent Interfaces

One of the ways to evolve the DTw is to add new services based on the available data. These services can leverage the wealth of contextualization and standardization of data collection to deliver more intelligence to meet user needs. Indeed, the arrangement of such standardized data allows standardization to be opened on the floors above the collection (interfaces, simulations, processing, etc.). Thus, the potential project benefits are potentially much greater. Funding requests can theoretically be larger. However, that is not the only benefit. The duplication of services and interfaces also allows for more user feedback, bugs, and possible improvements that will benefit all users. The involvement of line operators, engineers, and managers is crucial to achieving this digital transformation of the industry.

The contextualization of data provides new perspectives for intelligent services and interfaces. Additionally, all the contextualization data can be utilized by the different applications. Thus, the data can be retrieved automatically according to their type, according to a filter (geographical area, line number, plant name, etc.). All this wealth of contextualization opens doors for intelligent customers to process them to perform standardized tasks. Object discovery in the address space provides automatic modularity reconfiguration based on connected devices.

Furthermore, standardization has the potential to standardize human interfaces. Thus, human–machine interaction is simplified by a more advanced knowledge of the interface by all users. The interface with the machine is no longer a blocking point in the understanding of the system because it is understood and experienced by a wider range of people. Human expertise can then be expressed much more easily to detect and solve problems not managed by IT. The interface can even be in symbiosis with the user to work together.

#### 4.3.2. Integration of New Sensors

**Figure 9:**
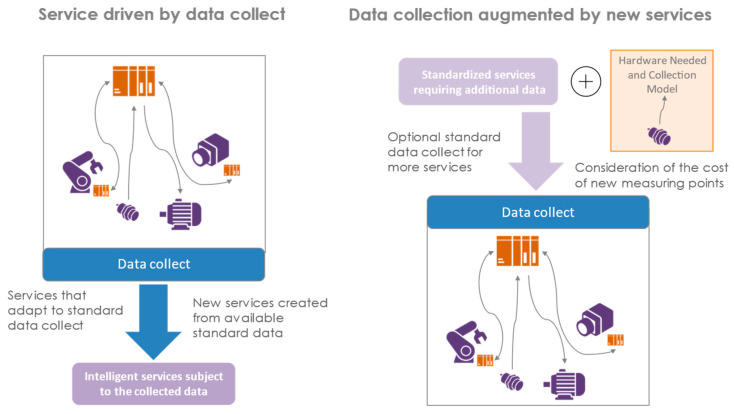
Data collection is the pivot between measurements and services.

Another way to evolve DTw is through the integration of new sensors to achieve new services. In this integrated approach from the sensor to the DTw, the service can be created by the sensors available through the collection of standardized and contextualized data, but the reverse is also possible. This is because a service may require the addition of standard sensors to the system. Thus, to implement this standard service, the additional sensors to be installed, as well as the collection model, are standardized. Thus, the standardization of a system does not end with the addition of additional sensors. On the contrary, it allows for a more in-depth study of the observability needs and the appropriate sensors to obtain the best result. The standardization of data collection can therefore be considered in the form of options to be able to integrate new services as needed. Additionally, smart services will be integrated directly if they are designed for the contextualized information of the new sensors. We see data collection as the pivot between the real and virtual worlds, requiring a two-way connection for connection but also for the integration of services and sensors into its architecture (Figure 9). The service has all the necessary data to work with the system’s basic data collection, or it will be necessary to add new measurement points. The hardware and the integration method are then standardized. Thus, the need is driven by data collection, and services are driven by available data. The material cost and the cost of implementing new measurement points must be able to measure the relevance of the implementation or its duplication.

#### 4.3.3. The Digital Twin at Scale

The reflections on the granularity of the data collected in the methodology aim to be able to carry out multiple instances of the DTw designed. This ability to deploy standardized data collection, as well as intelligent clients that can use contextualization metadata, provides an opportunity for large-scale deployment of DTws. It is a data-collectable-driven approach to the DTw for the manufacturing sector. The certain standardization of production equipment opens this opportunity in this field. As the DTw is a very expensive technology, this approach, by collecting data that can be used directly, complemented by simple services, is a first step in allowing training and the understanding of the possibilities and limitations of the DTw. The complexity and excitement around the word “Digital Twin” should warn decision-makers about projects that are too complex for the maturity of their business.

A standardized modularity of the collection allows the creation of a collection as close as possible to its needs as a DTw. In this approach, a DTw service could reconfigure (if it has permission) the data collection to automatically populate the address space of the variables it requires. Existing data will be collected once and contextualized for all needs. However, this possibility only exists if the information in the controller is standardized. In the opposite case, humans must intervene to map between variables.

**Figure 10:**
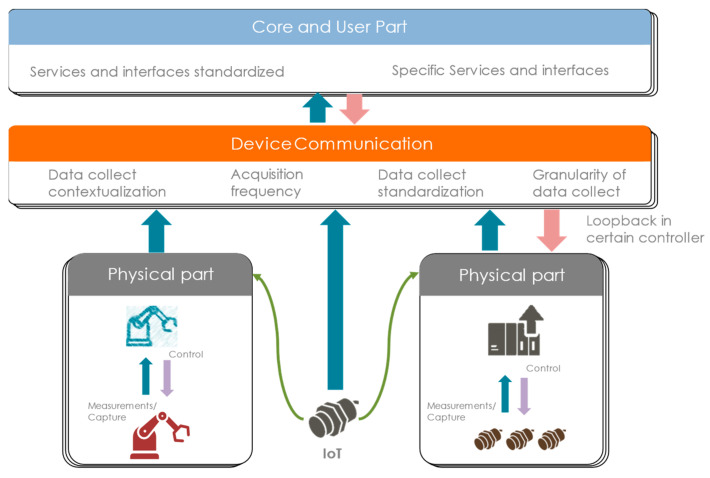
Sensors to Digital Twin.

Figure 10 synthesizes the different issues for the design of a DTw that can be scaled from the sensor to the services. This hybrid approach, based on the need and the possibilities of standardizable collection, offers the possibility of rapid and cost-effective mass deployments. This integrated approach from the sensor to the DTw is particularly intended for the manufacturing world, which is already loaded with sensors. The human element must not be forgotten in the design of the DTw and its evolution so as not to unnecessarily complicate the work of some people. This consideration allows for better acceptance of the DTw and better feedback from the field to augment the existing system and push the limits for future generations.

## 5. Discussion

The possibilities of transmitting data from sensors present for control or from the various IoT systems capturing the environment of a production line are not easy. Indeed, the network architectures of the manufacturing industry are particularly effective in preventing cyberattacks on their production. Additionally, the code of the PLCs controlling these lines is only, in very rare cases, standardized. In the case of a non-standardized controlling system, it is not possible to utilize a collection standard, including automatic mapping of variables to connect to the PLCs. Methodological work on the order therefore seems essential to deploy intelligent data collection and DTws on a large scale on manufacturing production lines.

This approach based on the connectivity of production line equipment seems promising to us for democratizing DTw implementations on a large scale and thus bringing research into the industrial world more quickly. However, this approach requires a common and unified effort by a group to deploy DTws and make them evolve together. This approach is applicable in cases where the same pattern is repeated. For unitary systems, the iterative approach is always relevant to limiting risks. However, it will only be instantiated once.

The collection of data from sensors on production lines should not only be intended for their use through DTws, but for all applications that need them. Thus, the collection is carried out once and only on the control, and made accessible securely to all authorized persons and applications. Better methodologies must be developed for each level of the DTw, both in terms of its design, implementation, and even interactions, but also to define the measurements to be collected, the controllable parameters, etc. It will be increasingly necessary to have methodologies in the design phase to have access to the desired information in the controllers. Indeed, IoT can integrate connectivity limitations to be able to sell and value the data it contains. Having methodologies and standards can make it possible to anticipate the problems of freedom of connectivity and thus choose the most appropriate technical solutions for the creation of smart factories augmented by DTws.

Increasing data collection is generating more and more data storage. It therefore seems important to know at least one use for the data collected before recording them. Questions then arise as to the lifespan of a piece of data, for which it is still relevant, but also the right times to collect it or what is the optimal way to store it.

## 6. Conclusions

The core of the contribution presented in this article is to propose a framework from an integrated approach of the sensor to the DTw with data collection as a central pivot. This architecture not only focuses on the importance and challenges of data collection, but also provides a rapid opportunity for large-scale deployment of DTws on common patterns. The iterative nature of the approach limits risk for DTw projects while providing scalability and flexibility. The same is true with the contextualization of data through a more explicit understanding of the data collected. This lower layer of the DTw linking the real and virtual worlds is difficult to implement on a large scale, especially as the system already exists with the aim of obtaining quality data. Through successive projects for the deployment of sensors and services, the DTw is maturing. Each project can be scaled to the available budget to continuously advance the DTw. Using contextualization data for service configuration makes it easier to maintain and scale the DTw. In addition, the integration of a new sensor brings with it all the services developed with the data available in the DTw.

As part of our future work, we plan not only to focus on control design methodologies but also on the realization of generic services for the DTw. The duplication of the DTw will allow its reliability and maintainability over time to augment systems. The design methodology proposed in this article offers an opportunity for a transition from today’s factory to tomorrow’s smart factory. This iterative approach smooths out effort, gains, and risks over time, from instrumentation to services and interfaces. The proposed approach will be applied in a real existing system in the near future.

## Figures and Tables

**Figure 1:**
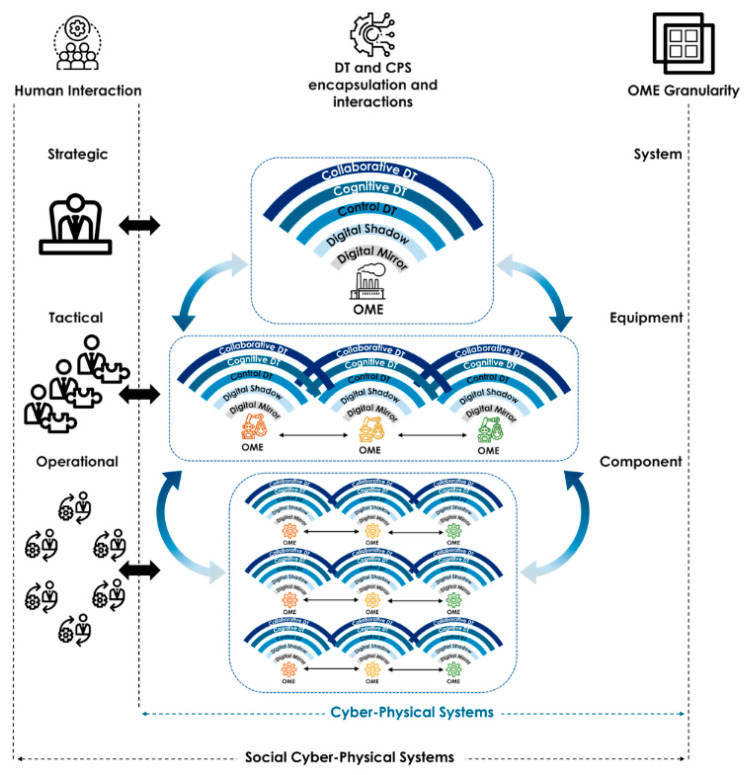
Hierarchical Digital Twin Network [26].

**Table 1:** Overview of domain-specific digital twin definition.

Author	Domain	Definition
Negrie et al. [24]	Manufacturer	“DT for manufacturing industry lies in their definition as virtual counterparts of physical devices. These are digital representations based on semantic data models that allow running simulations in different disciplines, that support not only a prognostic assessment at design stage (static perspective), but also a continuous update of the virtual representation of the object by a real time synchronization with sensed data. This allows the representation to reflect the current status of the system and to perform real-time optimizations, decision making and predictive maintenance according to the sensed conditions.”
ISO 23247 [25]	Manufacturer	“Manufacturing digital twin fit for purpose digital representation of an observable manufacturing element with synchronization between the element and its digital representation.”
Tuegel [27]	Aeronautics	“An ADT is a cradle-to-grave model of an aircraft structure’s ability to meet mission requirements. It is a submodel of an all-encompassing Aircraft Digital Twin which would include submodels of the electronics, the flight controls, the propulsion system, and other subsystems.”
Stark et al. [23]	Product	“A digital twin is a digital representation of an active unique product (real device, object, machine, service, or intangible asset) or unique product-service system (a system consisting of a product and a related service) that comprises its selected characteristics, properties, conditions, and behaviors by means of models, information, and data within a single or even across multiple life cycle phases.”
Talkhestani et al. [22]	Product	“The Digital Twin is a virtual model of a physical asset capable of fully mirroring its characteristics and functionalities during its entire lifecycle.”

## Data Availability

Data are contained within the article.
